# Trend of geographical distribution of stomach cancer in Iran from 2004 to 2014

**DOI:** 10.1186/s12876-021-02066-z

**Published:** 2022-01-04

**Authors:** Farid Moradian, Mohammad Fararouei, Maryam Karami, Mousa Ghelichi-Ghojogh, Zahra Gheibi, Zahra Nikeghbalian, Atieh Akbari, Mohammad-Esmaeil Akbari

**Affiliations:** 1 Cancer Research Center, Shahid Beheshti University of Medical Sciences, Tehran, Iran; 2 HIV/ADIS Research Center, School of Health, Shiraz University of Medical Science, Shiraz, Iran; 3 School of Nursing and Midwifery, Shahid Beheshti University of Medical Sciences, Tehran, Iran; 4 Student Research Committee, Shiraz University of Medical Sciences, Shiraz, Iran; 5 Department of Epidemiology, Shiraz University of Medical Science, Shiraz, Iran

**Keywords:** Stomach cancer, Gastric cancer, Geographical distribution, Incidence

## Abstract

**Background:**

Among different common types of cancer, gastric cancer (GC) is a worldwide health priority in both developing and developed countries. The aim of this study was to map the distribution of incident cases of GC in Iran to provide a geographical presentation of the incidence of the disease.

**Methods:**

This study used the Iranian National Cancer Registry (INCR) data from 2004 to 2014. We calculated the crude and age-standardized incidence rates of GC for each province and also defined the frequency distribution of different types and locations of GC by the provinces.

**Results:**

According to the results of the present study, the patients were predominantly male 49,907 (70.0%) and the most prevalent type of tumour was A1 (almost 96.4%) and C3‌ (2.0%). Also, a significant difference was observed between males and females in the distribution of the types of tumour (*P* < 0.001). In addition, a comparison of the distribution of the types of GC in Iran suggested that a significant difference exists between the provinces (*P* < 0.001). A significant difference was observed when the distribution of the location of GC tumors was compared between males and females and provinces (*P* < 0.001). Accordingly, pylori and cardia are the most common location of GC cancer among the study population (28.1% and 31.3% respectively).

**Conclusions:**

The results of the current study suggested a higher rate of GC incidence in Iran when compared to the global figure in both females and males. Our study also revealed significant disparities between provinces with regard to the distribution of types, and location of GC. This may suggest involving different factors in GC in different parts of Iran. Further studies are needed to better understand the epidemiology and etiology of the disease in Iran.

**Supplementary Information:**

The online version contains supplementary material available at 10.1186/s12876-021-02066-z.

## Background

According to a report (GLOBOCAN) in 2020, 1 in 5 people are diagnosed with cancer during their lifetime, and 1 in 8 men and 1 in 11 women die due to the disease [1]. The same report also ranks gastric cancer (GC) as the fifth leading cause of morbidity due to cancer (the third leading cause of death from cancer). As a result GC is a worldwide health priority in both developing and developed countries due to its fatal and nonfatal, but serious, health consequences [2]. It is estimated that each year, 1 089 103 new cases (5.6% of total new cases of cancer) and about 768,793 deaths (7.7% of total deaths due to cancer) due to stomach cancer occur in the world [1]. In addition, the incidence of GC is highly variable in different parts of the world. For example, the incidence of the disease is especially high in Oceania, North America and Europe respectively and very low in Africa [2]. This phenomenon may represent the potential differences in the etiology of GC in different regions [3]. In Iran, possibly due to the recent demographic and epidemiological transitions, the number of incident cases of gastric cancer is rising constantly [4]. This is why, currently, the incidence of gastric cancer is the first cause of death due to cancer in Iran [5]. Suggesting a large geographical discrepancy in Iran [6], it is reported that Ardebil (in the northeast of the country) has the highest incidence rate of GC when compared with different provinces in Iran and many parts of the world [4, 7].

With regard to the pathology and clinical aspects of GC, the anatomic appearance of cancer (cardia and non-cardia) and histological type of the disease (enteric and non-enteric) are two important characteristics of GC. These features may come with different etiology, symptoms, and even prognosis. According to a study, in different parts of Iran (i.e. Khorasan, Lorestan, Tehran, East-Azarbahijan, Sistan&Balochestan, Kurdestan, Mazandaran, and Khoozestan) distal gastric tumors are more common than proximal gastric tumors [8]. With regard to gender, GC is being more common among men [9]. However, Hae Won Kim suggested that Signet ring cell carcinoma (SRC) in GC was more prevalent among younger females than males [10]. Numerous studies are conducted to identify factors contributing to stomach cancer in different parts of the world and the results came out in favor of a wide range of factors including *H. Pylori* infection, dietary habits, physical inactivity, smoking, alcohol use, obesity, and family history of the disease, factors that are globally distributed with high discrepancy [11].

Due to the higher occurrence of stomach cancer in Iran, the issue has become of great national health concerns. As a result, mapping incident cases of GC and its important characteristics (location and histology) is a very informative step in better understanding the epidemiology of the disease in Iran. The aim of this study was to map the distribution of incident cases of GC in Iran to provide a geographical presentation of the incidence and pathology of the disease. The study also aimed to identify regions with unusually higher rate of GC in the country.

## Methods

During the last 3 decades, the Iranian National Cancer Registry (INCR) is founded and improved rapidly as a part of the National Health Care System. More information about NCR program in Iran is provided before. In summary, all cancer cases are to be confirmed by pathologic diagnosis and then to be registered with the NCR. As with other types of cancers, cases of GC are also pathologically confirmed and registered to the NCR database. This study used the NCR data from 2004 to the latest available data in 2014 (due to data cleaning, cancer database in Iran takes several years to became available for research proposes) to investigate the geographical distribution of the incidence of stomach cancer during the study period. Also, to see if there is any substantial differences between the provinces in the incidence, location and type of GC cases we also measured the rate of change in the incidence of GC by province (n = 31).

### Case definition

Uniformly in each province, the diagnosis of the GC cases is confirmed by a positive pathology report issued by a pathologist using microscopic verification method.

### Data source and preparation

The Iranian NCR is under the ministry of health and covers all provinces and all pathology centres in each province. Due to the large number of centres reporting cancer cases in each province, there is a high possibility of errors during data entry. As a result, data is to go under a long and rigorous checking, correction, and cleaning procedures. The procedures take about 5-6 years to make data ready for being published. In the current study, in addition to the routine checking of the data by NCR, the dataset was again quality controlled and rechecked for any duplicate cases or errors before being used for analysis.

### Calculation of Incidence rates

In Iran, censuses were conducted in the years 2006, 2011 and 2016. We calculated annual incidence rates based on the population size reported by Iranian centre for statistics (ICS). The annual incidence rates were calculated based on the reported incident of GC cases for each year divided by the nearest estimated population size for the years 2006,2011, and 2016. Due to the limited number of patients and prevent instability of the casuistry when studying incidence of cancer by gender-age, the ten-year incidence rates were used.

### Spatial and statistical data analysis

Excel (2015) was used to do the primary analysis including calculation of GC incidence for each province and also to provide a frequency table of the type and location of GC by the provinces. ArcGIS 10.5 (Redlands, CA, USA) mapping software was used to map the provincial age-standardized incidence rates by sex. Again, to prevent instability of the incidence rates, when they are presented by age and sex, we calculated the rates for the whole study period. The type and location of the GC cases were obtained from the NCR dataset. The rates were age-standardized using the methods and the standard population distribution that are provided by WHO [12]. The distribution of types and location of cancer by gender and province was tested by Chi-square. The randomness of the distribution of the cancer cases between the Iranian provinces was tested via Pearson’s chi-squared test statistic using R4.0.2 (prop.test function) The trend of incidence during the study period was tested using prop.trend.test function). All plots, GIS maps and figures were designed in GIS center, Department of Epidemiology, Shiraz University of Medical Sciences.

## Results

According to the results of the present study, the patients were predominantly male (70.0%) and were predominantly of older age (mean = 65.9, SD = 14.7). The 10-year incidence rates of stomach cancer by age and sex are presented in Table 1 and Additional file 1, 2: Figs. S1, S2. The incidence of GC in Iran for 2014 (13.0 × 10^5^) is larger than the corresponding global rate (12.1 × 10^5^ respectively) [13]. In addition, although the age-specific incidence rate of GC is rising by age in both genders, GC starts earlier and raises faster in men compared to women.

**Table 1:** The 10-year incidence of stomach cancer by age, sex

Age group	Men			Women			Total
	Cases	Population	Incidence/100,000	Cases	Population 2014	Incidence/100,000	Incidence/100,000
[0–5)	7	3,459,169	0.0	0	3,274,638	0.0	0.0
[5–10)	2	3,123,814	0.0	1	2,971,835	0.0	0.0
[10–15)	3	2,895,688	0.0	0	2,773,242	0.0	0.0
[15–20)	5	3,004,634	0.0	8	2,902,202	0.0	0.0
[20–25)	25	3,616,279	0.1	19	3,571,851	0.0	0.1
[25–30)	55	4,218,536	0.1	35	4,151,876	0.1	0.1
[30–35)	80	3,999,442	0.2	69	3,927,181	0.2	0.2
[35–40)	111	3,273,844	0.3	110	3,158,103	0.3	0.3
[40–45}	205	2,675,355	0.7	134	2,583,994	0.5	0.6
[45–50)	304	2,277,422	1.2	171	2,222,709	0.7	1.0
[50–55)	519	1,886,531	2.5	223	1,870,494	1.1	1.8
[55–69)	718	1,528,642	4.3	304	1,546,018	1.8	3.0
[60–65)	797	1,101,117	6.6	404	1,164,036	3.2	4.9
[65–70)	882	740,984	10.8	409	819,830	4.5	7.7
[70–75)	919	565,960	14.8	394	586,293	6.1	10.4
[75–80)	1050	461,003	20.7	432	434,622	9.0	14.9
[80–85)	842	317,822	24.1	339	293,916	10.5	17.3
85+	536	220,979	22.1	265	209,044	11.5	16.8
Total	7060	39,367,223.6	17.9	3317	38,461,884.2	8.6	13.3

**Fig. 1:**
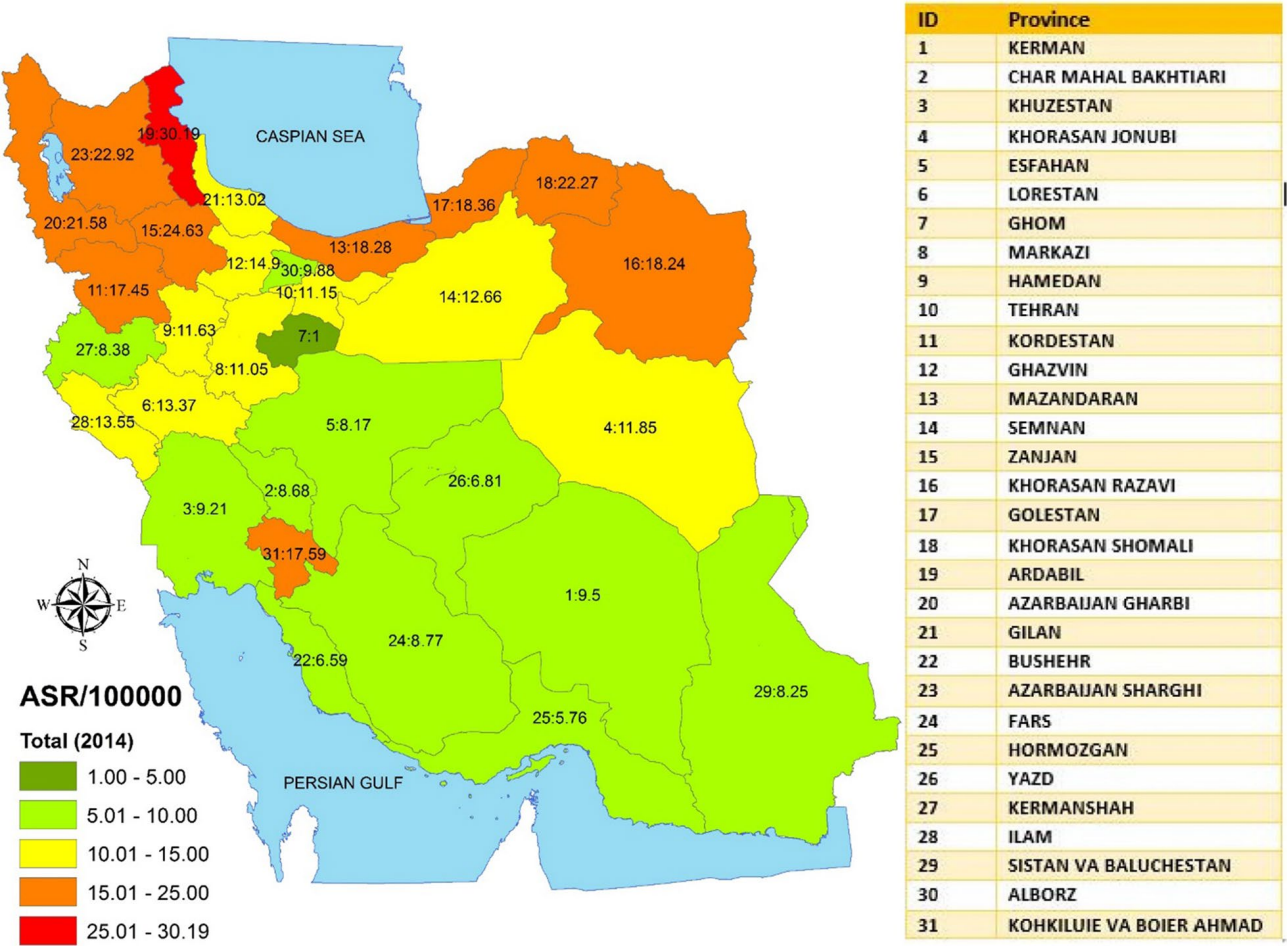
Distribution of ASR of gastric cancer by province in Iran in 2014 (both genders) (GIS map were designed in GIS center, Department of Epidemiology, Shiraz University of Medical Sciences)

**Fig. 2:**
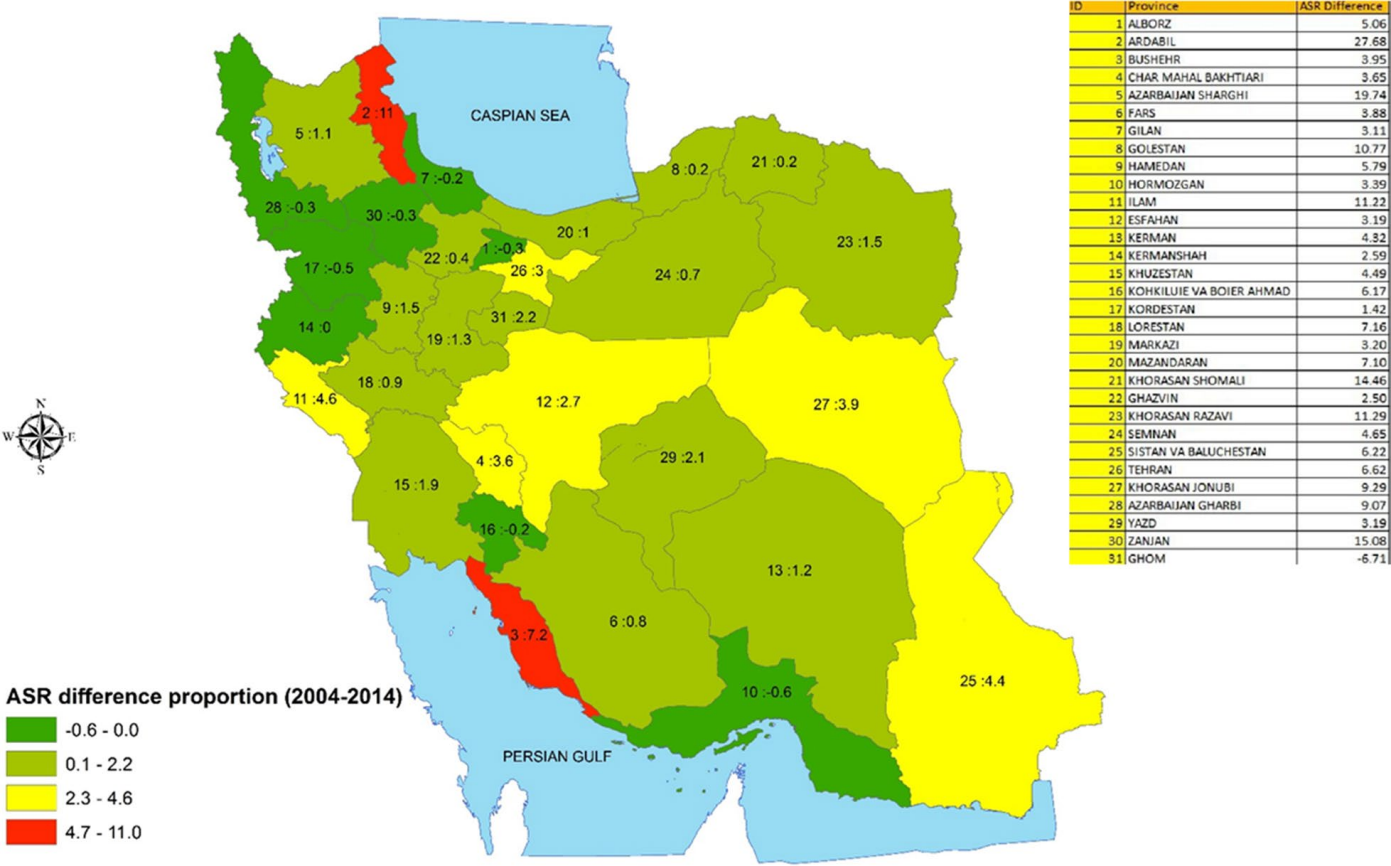
Rate of change in the incidence of gastric cancer from 2004 to 2014 by province (GIS map were designed in GIS center, Department of Epidemiology, Shiraz University of Medical Sciences)

During the study period, the highest and lowest age-standardized incidence rates of GC in Iran in 2014 were reported from Ardebil (ASR = 30.19) and Ghom (ASR = 1.00) respectively (Fig. 1). With regard to the rate of change of the GC, in general, the rate of stomach cancer was rising during the study period (5.63 in 2004 to 13.33 in 2014, 136.65% increase, *P* for trend < 0.001). The highest rates of change (from 2004 to 2014) were also observed from Ardebil (1100% increase) and Ghom (30% decrease) respectively (Fig. 2).

**Fig. 3:**
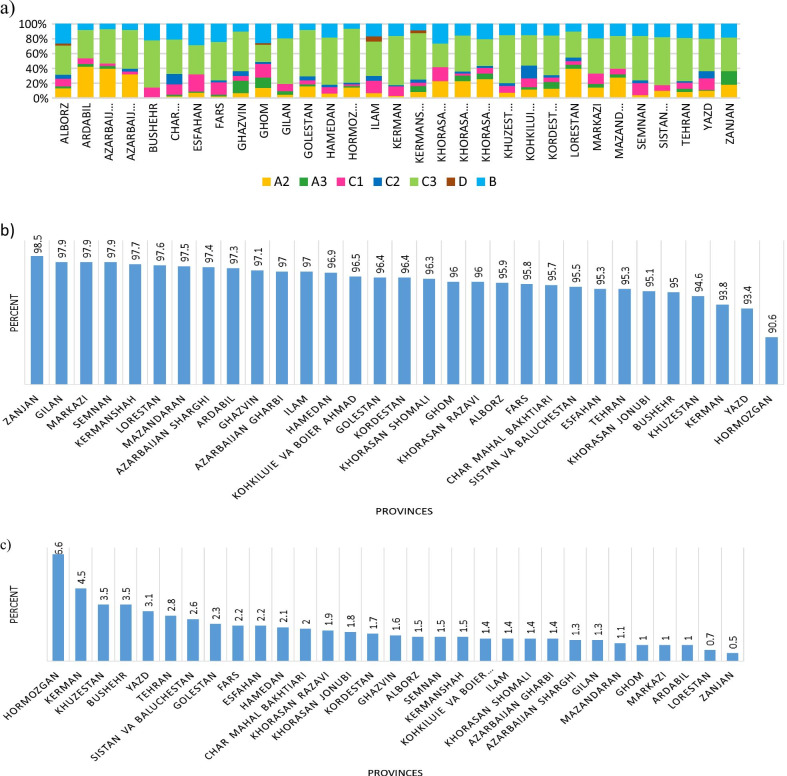
The distribution of M codes of gastric cancer by province. **a** all m-codes (A1 M-code is excluded due to its very large proportion); **b** A1 M-code; **c** C3 M-code. A1: Adenocarcinoma, A2: Squamous Cell Carcinoma, A3: Others. B) Neuroendocrine Tumors; B1) Well Differentiated Neuroendocrine Tumors, B2) Neuroendocrine Carcinomas. C) Non Epithelial Tumors; C1) GIST, C2) Sarcoma, C3) Lymphoma. D)Metastasis

**Fig. 4:**
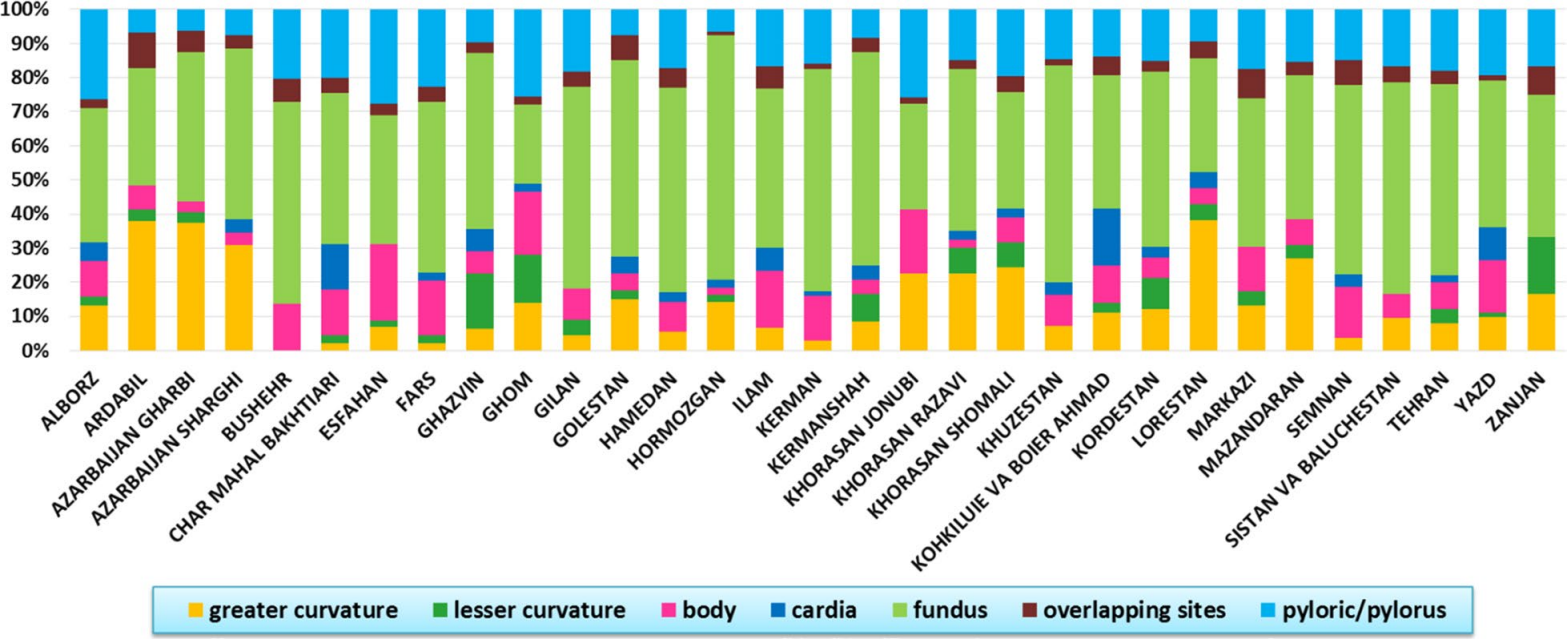
Location of gastric cancer by province

With regard to the type of tumour, the most prevalent types of tumour were A1 (almost 96.4%) and C3‌ (2.0%). Also, a significant difference was observed between males and females in the distribution of the types of tumour (*P* < 0.001). In addition, a comparison of the distribution of the types of GC in Iran suggested that a significant difference exists between the provinces (Fig. 3, *P* < 0.001). Accordingly, the highest rate of A1 type of GC cancer was observed in Zanjan, (98.5%) (Fig. 4). Whereas, the highest rate of C3, (the second most common type of GC cancer among the Iranian population) was observed in Hormozgan (6.6%) (Additional file 3: Fig. S3). With regard to the location of the tumor, a significant difference was observed between the two genders (*P* < 0.001). Accordingly, in males, cardia and fundus were the most common locations of GC tumors whereas in females, pylorus was the most common location of GC tumors in the study population (Additional file 4: Fig. S4).

According to our study results, pylori and cardia are the most common location of GC cancer among the study population (28.1% and 31.3% respectively). Comparing the location of GC between provinces, Azarbaijan Sharghi and Bushehr had the highest and lowest rates of fundus-based GC (Fig. 4 and Additional file 3: Fig. S3). With regard to the tumours located in pylori, Hoemozgan and Azarbaejan Sharghi were the provinces with the highest and lowest figures (Fig. 4 and Additional file 5 Fig. S5, *P* < 0.001).

## Discussion

The yet growing national incidence rate of GC is already higher than its corresponding global figure. The sex-age-specific incidence rates of GC suggested a clear difference between males and females as GC starts much earlier (even during childhood) in men when compared to women. A traditional explanation for such difference between the two genders is the higher rate of smoking and alcohol use in men than women. However, according to the results of the present study, GC in men is started at younger ages, when exposures to these factors are rare (we don’t expect much smoking or alcohol drinking habits among Iranian children or adolescents) and too close to the outcome (no temporality). On the other hand, our study results suggested that GC starts rising sharply in men (compared to women) at about 40 years of age, an expected time for seeing the causal actions of smoking, alcohol drinking, and drug use in men. According to the results of the current study, it seems that GC among the Iranian population is driven by different factors at different ages.

Since GC is an important and growing multifactorial health issue in Iran, defining the spatial distribution of the disease is of utmost importance. According to the results of our spatial analysis, Ardebil had the highest rate of incidence and the highest rate of raise in the incidence of GC. The province also come with the highest age-standardized incidence rates in both men and women. Previous studies suggested that the high incidence of esophageal cancer in this province (Ardebil) could be due to the presumed belt for upper gastrointestinal tract cancers. Including stomach and esophagus cancers, the belt is originated from the Far East or East Asia and crosses Central Asia and Near East, the geographical location of Iran [14]. It is worth noticing that the distribution of esophageal cancer in Iran is also highly variable. The results of the present study also showed that Ghom had the lowest incidence rate of stomach cancer during the study period. Our estimated rate of change during the study period suggests that all provinces in the northern part of the country were among the provinces with the higher rates of incidence but with a relatively low rate of change in the incidence of GC. The only exception was observed for Ardabil (the province with the highest rates of both incidence and change in the country). The observed upward trend in the incidence rate of GC in Arak (an industrial province in the center of the country) is in accordance with what was reported by Moradzadeh who used local data [15]. The observed patterns of change in the incidence of GC during the study period (10 years) suggests that despite having a relatively higher annual rate of GC incidence, a lower rate of change (raise) is observed (except for Ardabil and Bushehr) in different parts of the country (the rate of change was even negative for Hormozgan, Kordestan, Zanjan Azarbaijan gharbi,, Alborz, Gilan, and Kohkilooei and Boirahmad). This finding suggests a gradual improvement in the affecting factors in these regions. On the other hand, the results of our study revealed that the incidence of GC is rising alarmingly in the central parts of the country, suggesting a steady raise in the effect of factors affecting GC in Iran.

With regard to the geographical distribution of the type of GC, the results of the present study showed that in Iran, different types of gastric cancer are observed with a highly significant intra-country variation. For example, when compared to the other Iranian provinces, the highest and lowest percentages of A1 type of GC were reported from Zanjan and Hormozgan respectively. The reason for this discrepancy in Iran is possibly to be found in a wide range of differences in the environment-genetic interaction, ethnicity, and lifestyle (especially dietary patterns) [14].

With regard to the location of GC tumors, the results of our study revealed that cardia is the most common location of GC among Iranian patients. This observation was also reported globally as according to WHO, gastric cardia cancer was responsible for 49.5% of all GC cases [16]. The results of the present study also suggested that pillory was the most common location of GC tumor in Iranian women whereas, in men, fundi was more common. Also, our study suggested that the location of GC is more common in cardiac than the other parts of the stomach in the northern provinces, whereas pylorus is the more common location of GC in the southern part of the country. The distribution of the location of GC in men and women is significantly different suggesting a partial difference between the two genders in factors determining the location of cancer in the stomach. These findings may also help us to understand the differences in the etiology of GC between the two genders and different countries. In that regard, in a meta-analysis, Abdulrazak reported that Helicobacter pylori infection is more frequent among men than women [17]. The difference between the two genders may also indicate the involvement of hormonal differences, behavioral related factors (e.g. smoking, alcohol, and drug use), and occupational exposures. For example, it is reported that lifestyles such as physical inactivity and obesity play important roles in the risk of cardiac gastric cancer. It is also suggested that sturgeon in women may protect them against the progression of gastric cancer [11]. Accordingly, delayed menopause and increased fertility may decrease the risk of gastric cancer among females [11]. The reported differences in the regional distribution of some risk factors may explain the observed geographical differences and trends of many types of cancer [14]. In that regard, Norouzinia reported that in different parts of Iran (Khorasan, Lorestan, Tehran, East-Azarbaijan, Sistan&Balochestan, Kurdestan, Mazandaran, and Khuzestan) the majority of tumors were distal gastric. They also suggested that many factors such as environmental, lifestyle, and ethnicity in different geographical locations may contribute to the overall incidence and the anatomical location of GC [8]. For example, variation in dietary patterns and certain cooking methods including broiling of meats, roasting, grilling, sun drying, and curing may explain why the risk of GC and the distribution of its types and locations are highly vary in different regions of Iran [16, 18]. Accordingly, the upward trend in GC in the northern part of Iran could be due to particular food combination (e.g. fiber, dairy foods and meat) and preparation methods (e.g. boiling, roasting or smoked) [19].

## Limitations

The cancer registry in Iran is fast improving. However, several important concerns regarding the validity and reliability of the Iranian NCR are raised [20]. The registry flaws may affect our understanding of the epidemiology of GC cancer in Iran. We were not able to make any etiologic inference on the observed differences between the Iranian provinces due to a lack of national data on major previously mentioned contributing factors such as H. pylori infection and diet of the study population. We could not include very important information such as cancer stage and the histopathological type of the tumors in our analysis as the Iranian NCR does not provide this information.

## Conclusion

The results of the current study revealed a higher rate of GC incidence in Iran when compared to the global figure in both females and males. Moreover, despite the downward trend of stomach cancer globally, our study suggested a significant rise in the incidence of the disease in Iran. The results also suggested significant discrepancies between provinces in the incidence of GC and also the type and location of the tumors. We need further rigorous studies on the involvement of different factors and their interactions in GC among the Iranian population. In addition, further studies on the nationwide distribution of other cancers of the digestive system may help us in finding the major players in the etiology of these types of cancer in Iran.

## Supplementary Information

**Additional file 1: Fig. S1**. Age-standardized incidence rates of gastric cancer by province in 2014 (females) (GIS map were designed in GIS center, Department of Epidemiology, Shiraz University of Medical Sciences).

**Additional file 2: Fig. S2**. Age-standardized incidence rates of gastric cancer by province in 2014 (males) (GIS map were designed in GIS center, Department of Epidemiology, Shiraz University of Medical Sciences).

**Additional file 3: Fig. S3**. Distribution of gastric cancer by tumour location.

**Additional file 4: Fig. S4**. Distribution of the location of gastric cancer tumors by gender.

**Additional file 5: Fig. S5**. Distribution of tumour located in pylori by provinces

## Data Availability

All relevant data are within the paper and its supporting information files.There is no separate data set to share.

## Abbreviations

EMRO: Eastern mediterranean region
GC: Gastric cancer
INCR: Iranian National Cancer Registry
SRC: Signet ring cell carcinoma

## References

[1] Sung H, Ferlay J, Siegel RL. Global cancer statistics 2020: GLOBOCAN estimates of incidence and mortality worldwide for 36 cancers in 185 countries. 2021. 10.3322/caac.21660 33538338

[2] Ferlay J, Colombet M, Soerjomataram I, Parkin DM, Piñeros M, Znaor A, et al. Cancer statistics for the year 2020: an overview. Int J Cancer. 2021. 10.1002/ijc.33588 33818764

[3] Esmaeimzadeh N, Salahi-Moghaddam A, Khoshdel A (2015). Geographic distribution of important cancers in Iran. Hormozgan Med J.

[4] Fattahi N, Moghaddam SS, Rezaei N, Rezaei N, Fattahi E, Moradveisi B, et al. The national trend of the gastric cancer burden in Iran from 1990 to 2017. Asia-Pacific J Clin Oncol. 2021. 10.1111/ajco.13563 33629817

[5] Hasanzadeh J, HosseiniNezhad Z, Molavi-eVardanjani H, Farahmand M (2013). Gender differences in esophagus, stomach, colon and rectum cancers in Fars, Iran, during 2009-2010: an epidemiological population based study. J Rafsanjan Univ Med Sci.

[6] Asmarian N, Jafari-Koshki T, Soleimani A, Ayatollahi SMT (2016). Area-to-area poisson kriging and spatial bayesian analysis in mapping of gastric cancer incidence in Iran. Asian Pacific J Cancer Prevent.

[7] Pakzad R, Khani Y, Pakzad I, Momenimovahed Z, Mohammadian-Hashejani A, Salehiniya H (2016). Spatial analysis of stomach cancer incidence in Iran. Asian Pacific J Cancer Prevent.

[8] Norouzinia M, Asadzadeh H, Shalmani HM, Al Dulaimi D, Zali M (2012). Clinical and histological indicators of proximal and distal gastric cancer in eight provinces of Iran. Asian Pacific J Cancer Prevent.

[9] Fararouei M, Parisai Z, Farahmand M, Haghighi RE, Toori MA (2015). Cancer incidence appears to be rising in a small province in Islamic Republic of Iran: a population-based cohort study. Eastern Mediterranean Health J.

[10] Kim HW, Kim J-H, Lim BJ, Kim H, Kim H, Park JJ (2016). Sex disparity in gastric cancer: female sex is a poor prognostic factor for advanced gastric cancer. Ann Surg Oncol.

[11] Karimi P, Islami F, Anandasabapathy S, Freedman ND, Kamangar F (2014). Gastric cancer: descriptive epidemiology, risk factors, screening, and prevention. Cancer Epidemiol Prevent Biomark.

[12] Ahmad OB, Boschi-Pinto C, Lopez AD, Murray CJ, Lozano R, Inoue M. Age standardization of rates: a new WHO standard. Geneva: World Health Organization. 2001;9(10).

[13] Ferlay J, Soerjomataram I, Dikshit R, Eser S, Mathers C, Rebelo M (2015). Cancer incidence and mortality worldwide: sources, methods and major patterns in GLOBOCAN 2012. Int J Cancer.

[14] Khazaei S, Ayubi E, Mansori K, Gholamaliee B, Khazaei S, Shadmani FK, et al. Geographic, sex and age distribution of esophageal cancer incidence in Iran: a population-based study. Middle East J Cancer. 2017;8(2).

[15] Moradzadeh R, Anoushirvani AA (2020). Trend of gastric cancer incidence in an area located in the center of Iran: 2009–2014. J Gastrointestinal Cancer.

[16] Pourfarzi F, Whelan A, Kaldor J, Malekzadeh R (2009). The role of diet and other environmental factors in the causation of gastric cancer in Iran—a population based study. Int J Cancer.

[17] Ibrahim A, Morais S, Ferro A, Lunet N, Peleteiro B (2017). Sex-differences in the prevalence of Helicobacter pylori infection in pediatric and adult populations: systematic review and meta-analysis of 244 studies. Digest Liver Dis.

[18] Wogan GN, Hecht SS, Felton JS, Conney AH, Loeb LA, editors. Environmental and chemical carcinogenesis. In: Seminars in cancer biology. Elsevier. 2004. 10.1016/j.semcancer.2004.06.010 15489140

[19] Babaei M, Pourfarzi F, Yazdanbod A, Chiniforush MM, Derakhshan MH, Mousavi SM (2010). Gastric cancer in Ardabil, Iran—a review and update on cancer registry data. Asian Pac J Cancer Prev.

[20] Fararouei M, Marzban M, Shahraki G (2017). Completeness of cancer registry data in a small Iranian province: a capture–recapture approach. Health Inf Manag J.

