# Supplementary material for: Trend of geographical distribution of stomach cancer in Iran from 2004 to 2014
Source: BMC Gastroenterol. 2022 Jan 4;22:4. doi: 10.1186/s12876-021-02066-z (PMC8725466; doi:10.1186/s12876-021-02066-z)
Supplement: Supplementary file 1 — Additional file 1: Fig. S1. Age-standardized incidence rates of gastric cancer by province in 2014 (females) (GIS map were designed in GIS center, Department of Epidemiology, Shiraz University of Medical Sciences). [file 12876_2021_2066_MOESM1_ESM.docx]

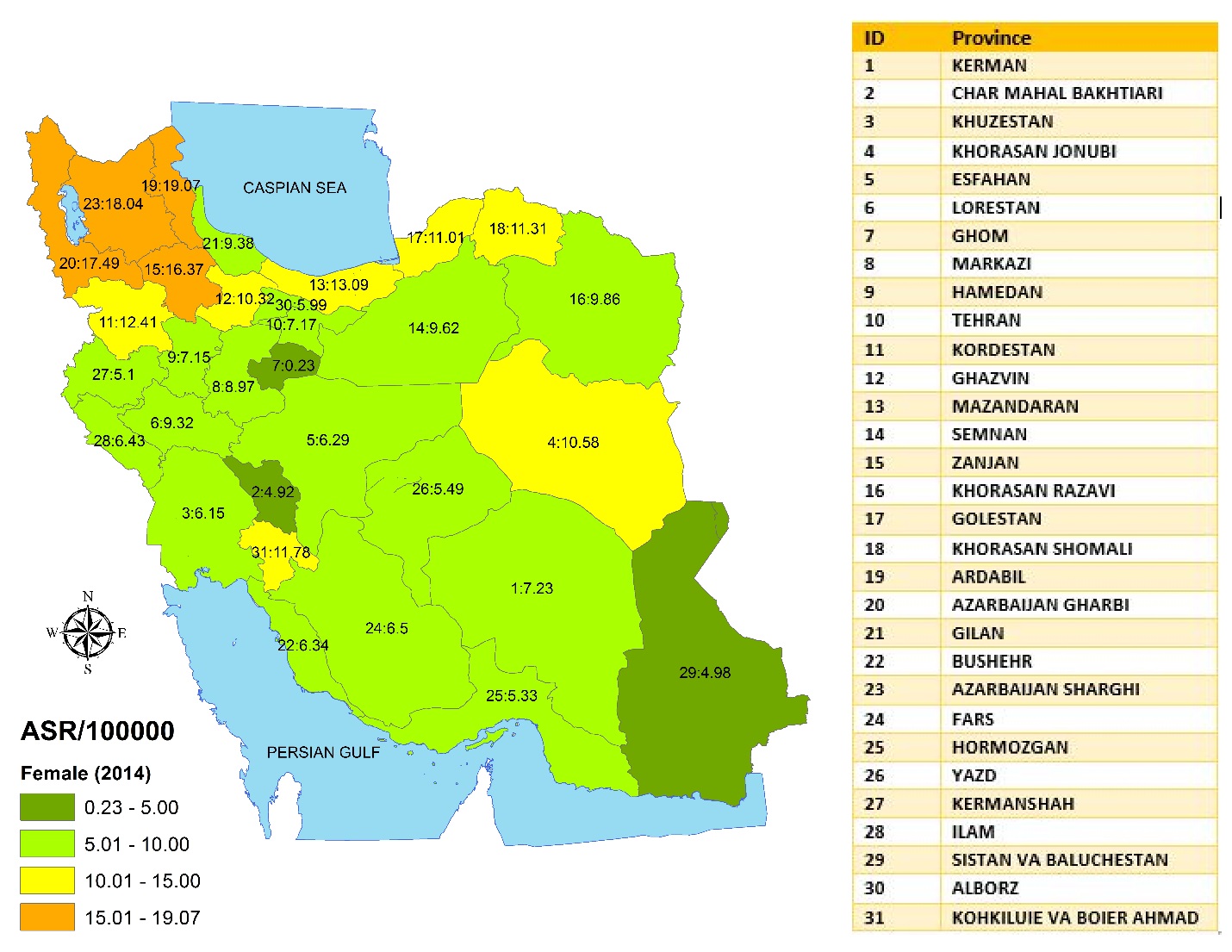


Supplementary Figure 1: Age-standardized incidence rates of gastric cancer by province in 2014 (females) (GIS map were designed in GIS center, Department of Epidemiology, Shiraz University of Medical Sciences)
