# Supplementary material for: Trend of geographical distribution of stomach cancer in Iran from 2004 to 2014
Source: BMC Gastroenterol. 2022 Jan 4;22:4. doi: 10.1186/s12876-021-02066-z (PMC8725466; doi:10.1186/s12876-021-02066-z)
Supplement: Supplementary file 3 — Additional file 3: Fig. S3. Distribution of gastric cancer by tumour location. [file 12876_2021_2066_MOESM3_ESM.docx]

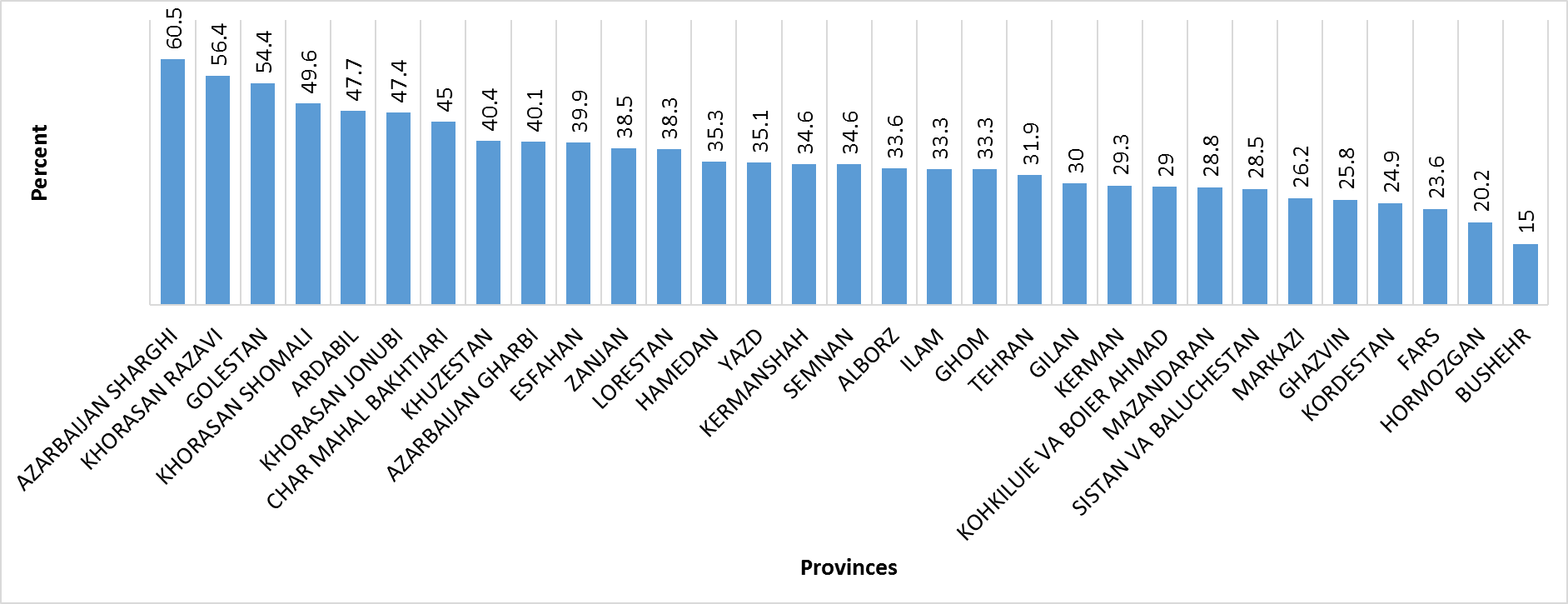


a) Distribution of tumors located in cardia by province


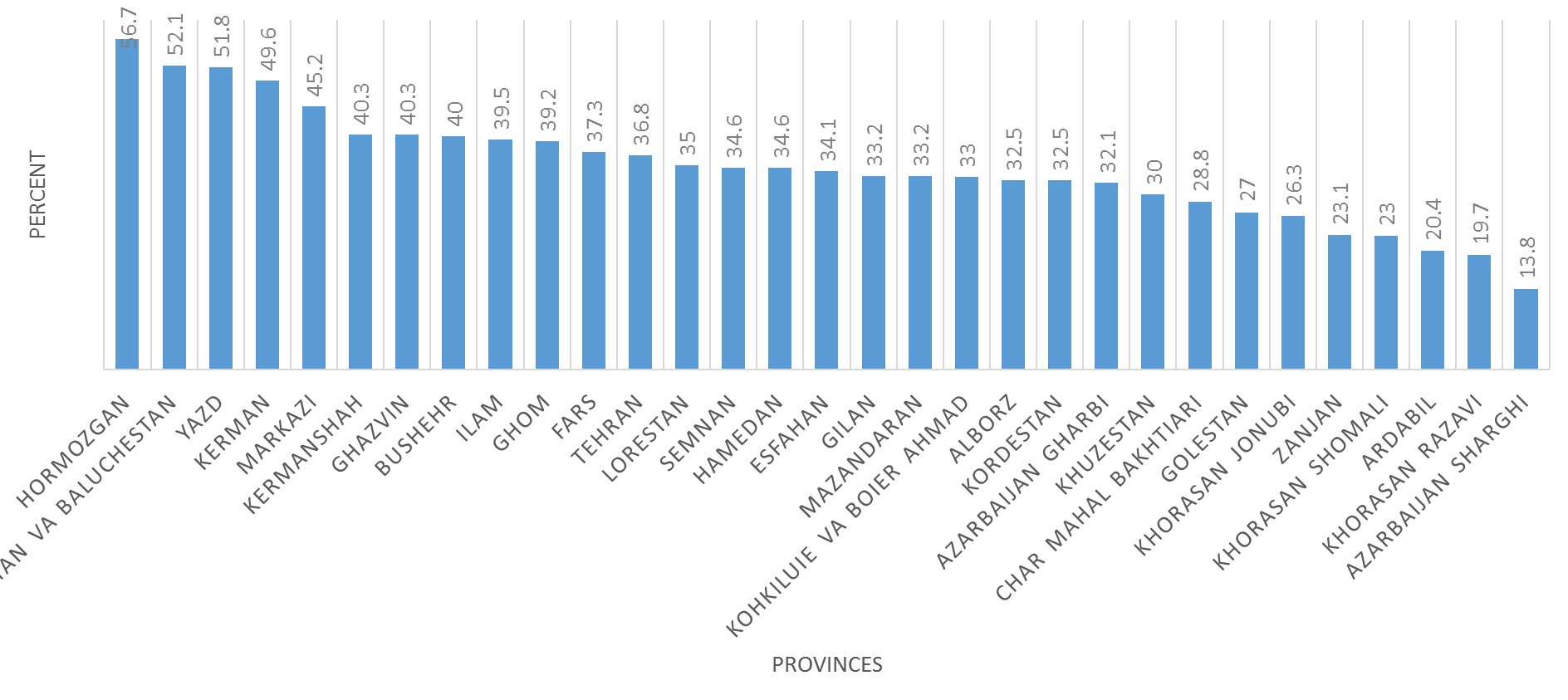


b) distribution of tumour located in pylori by province

Supplementary Figure 3: Distribution of gastric cancer by tumour location
