# Supplementary figures and images for: Trend of geographical distribution of stomach cancer in Iran from 2004 to 2014
Source: BMC Gastroenterol. 2022 Jan 4;22:4. doi: 10.1186/s12876-021-02066-z (PMC8725466; doi:10.1186/s12876-021-02066-z)

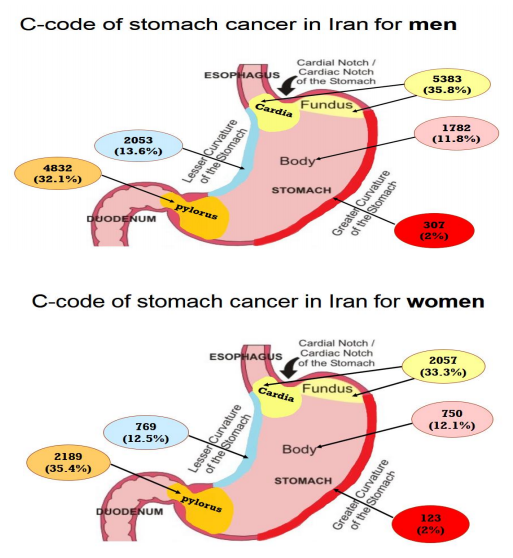


Supplementary Figure 4: Distribution of the location of gastric cancer tumors by gender

Supplement: Supplementary file 4 — Additional file 4: Fig. S4. Distribution of the location of gastric cancer tumors by gender. [file 12876_2021_2066_MOESM4_ESM.docx]

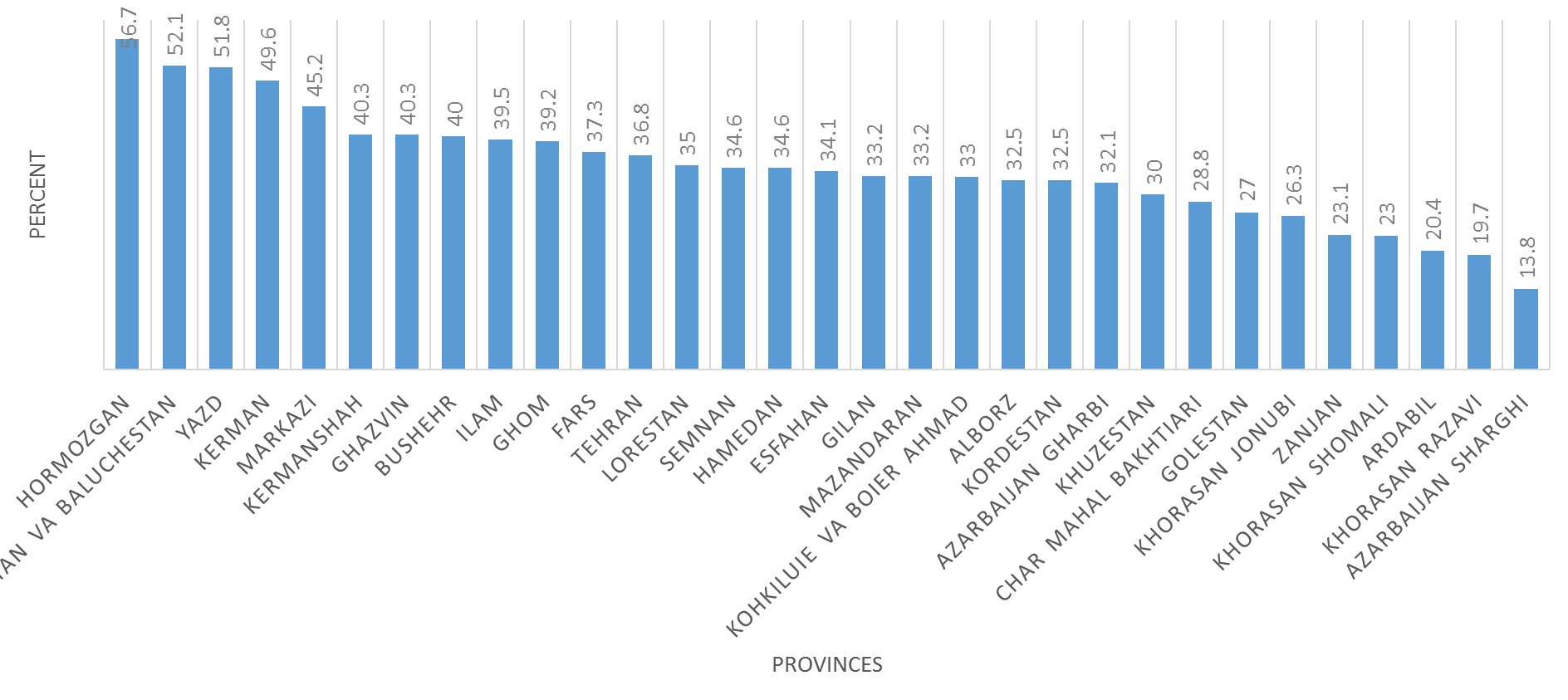


Supplementary Figure 5: distribution of tumour located in pylori by provinces

Supplement: Supplementary file 5 — Additional file 5: Fig. S5. Distribution of tumour located in pylori by provinces [file 12876_2021_2066_MOESM5_ESM.docx]
